# Case Report: Subcutaneous Emphysema and Pneumomediastinum Following Dental Extraction

**DOI:** 10.5811/cpcem.2020.9.49208

**Published:** 2021-01-11

**Authors:** Ryan M. Brzycki

**Affiliations:** Mercy St. Vincent Medical Center, Department of Emergency Medicine, Toledo, Ohio

**Keywords:** Pneumomediastinum, subcutaneous emphysema, dental procedure, dysphagia, dental emergency

## Abstract

**Introduction:**

Emergency physicians should be cognizant of complications following common procedures (including dental) and be able to readily care for patients with acute dental pain.

**Case Report:**

A 22-year-old female presented with dental pain and difficulty swallowing that developed 48 hours after she underwent a dental extraction. The physical exam showed an uncomfortable, afebrile female with dysphonia, inability to tolerate secretions, and crepitus over the neck and anterior chest wall.

**Discussion:**

The use of a high-speed dental drill may have caused air to dissect through fascial planes leading to subcutaneous emphysema, or even through deeper planes resulting in pneumomediastinum. It should be noted that subcutaneous emphysema and pneumomediastinum are rare complications of dental procedures.

**Conclusion:**

This case highlights an uncommon but potentially life-threatening complication following a routine dental procedure, which emergency clinicians should be attentive to and able to identify and thereby manage.

## INTRODUCTION

Subcutaneous emphysema and pneumomediastinum have a myriad of etiologies ranging from trauma or infection to post-surgical or even spontaneous origin.^1^ Although these complications may arise following innocuous dental procedures such as tooth extraction, they are far more rare.^1^,^2^ When subcutaneous emphysema and pneumomediastinum do occur following dental procedures, they are almost always a result of the use of a compressed air drill and involve the mandibular molars.^1^ This case highlights the rare complication of subcutaneous emphysema and pneumomediastinum following a tooth extraction with local anesthetic. The mechanisms, clinical presentations, complications, and management are reviewed.

## CASE REPORT

A 22-year-old female presented to the emergency department (ED) with the complaint of left-sided jaw pain, left cheek swelling, dysphagia, and odynophagia that developed 48 hours after a dental extraction. The patient had been seen in the ED earlier that day for left jaw pain but did not endorse odynophagia at that time and was capable of eating and drinking. She denied any fever, headache, vision change, eye pain, nausea, vomiting, dyspnea, or use of straws. Her past medical history did not contain any pre-existing cardiopulmonary disease and she was in good health without any prior surgeries. She denied tobacco or alcohol use. Two days prior to her visit to the ED, she underwent a tooth extraction at her dentist’s office where her left mandibular first molar was removed. The patient received bupivacaine with epinephrine injection for local anesthesia. Later review of dental records indicated that a pressurized air turbine dental drill had then been used for resection of the tooth prior to extraction.

Her vital signs in the ED were as follows: blood pressure of 124/68 millimeters of mercury; heart rate 82 beats per minute; respiratory rate 15 breaths per minute; oxygen saturation of 98% on room air; and oral temperature 36.8° Celsius. Physical examination showed an uncomfortable-appearing female who was expectorating saliva into an emesis basin. She exhibited slight dysphonia but no stridor, and was able to speak in full sentences without difficulty. The patient exhibited tenderness to palpation over the left mandible with mild cheek edema, without erythema. Partial dislodgement of blood clot overlying the left first mandibular molar (tooth number 19) was noted. The trachea was midline and there was no meningismus, although she had increased midline neck pain with resistance to neck extension (secondary to pain). She had palpable crepitus over the neck and anterior chest wall.

Laboratory findings showed a white blood cell count of 6.8 thousand per microliter (K/uL) (reference range 3.5–11.3 k/uL), hemoglobin of 15.2 grams per deciliter (g/dL) (ref range 11.9–15.1 g/dL), platelet count of 300 (K/uL) (ref range 138–453 K/uL), and C-reactive protein 8 milligrams per liter (mg/L) (ref range < 5 mg/L). A computed tomography (CT) soft tissue neck with intravenous (IV) contrast was performed, which demonstrated extensive air accumulation involving the oral, retropharyngeal space, and pneumomediastinum (Image). After consultation with a cardiothoracic surgeon, the decision was made to begin prophylactic antibiotics. She was started on IV maintenance fluids and ampicillin-sulbactam for broad spectrum coverage including oral flora.

The patient was admitted for observation and discharged three days later. An esophagram taken to rule out esophageal damage showed that the esophagus appeared to be without injury and was functioning normally. The patient received a follow-up phone call one week after being discharged and reported complete resolution of symptoms.

**CPC-EM Capsule**

What do we already know about this clinical entity?

Subcutaneous emphysema and pneumomediastinum following dental procedures is a rare but life threatening complication that may be easily overlooked.

What makes this presentation of disease reportable?

Emergency physicians should be aware of the rare but serious complications that may occur following seemingly innocuous dental procedures.

What is the major learning point?

The images serve to solidify the severity of the disease process that may occur following local dental extraction.

How might this improve emergency medicine practice?

When a patient presents with dental pain, dysphagia, or odynophagia following dental extraction, allergic reaction and subcutaneous emphysema with pneumomediastinal extension should be considered.

## DISCUSSION

Emergency physicians are more likely to encounter subcutaneous emphysema and pneumomediastinum from non-dental causes. These non-dental causes include traumatic intubation, mechanical ventilation, facial trauma, forceful vomiting leading to esophageal rupture, asthma exacerbation with alveolar rupture, and intense Valsalva maneuver. They can also result following endoscopic procedures such as tracheostomy, head, neck or thoracic surgery, endoscopy, bronchoscopy or, rarely, foreign body or tumor in the bronchopulmonary tree or digestive tract.^1^ Subcutaneous emphysema with pneumomediastinal extension are also rare complications following various dental procedures, especially after the use of an air pressurized dental drill used for cutting, extracting, and cooling dental surfaces.^1^

When a pressurized drill is overused or used at an improper angle, it forces pressurized air and unsterile water beneath soft tissue spaces via disruptions in the dentoalveolar membrane.^2^–^4^ Air and water may then dissect along the multiple fascial planes between the mouth and mediastinum, especially near the roots of the three molars that directly communicate with the sublingual and retropharyngeal spaces, risking the spread of contaminants from the gingival flora into the mediastinum.^2^ Mediastinal extension is associated with a myriad of potentially serious complications that increase morbidity and mortality such as infective mediastinitis, tension pneumothorax, pericardial tamponade, airway obstruction, or even air embolism.^2^,^5^–^7^

Physicians might attribute immediate dyspnea and swelling after a dental procedure to allergic reaction or angioedema, and delayed symptoms to hematoma or soft tissue infection such as cellulitis, Ludwig’s angina or Lemierre’s syndrome.^5^,^8^ Patients with isolated subcutaneous emphysema typically present with painless edema of the face and neck; however, the presence of palpable crepitus is pathognomonic and clearly distinguishes from other causes.^4^,^5^,^9^ Symptom onset is within a few hours following the procedure in over 90% of cases, but seldomly beyond 48 hours.^6^,^11^ Rarely, dysphonia, dyslalia, brassy voice, and hearing loss occur due to free air in the retropharyngeal space compressing the Eustachian tube.^8^ Once palpable crepitus is noted, there should be immediate consideration for pneumomediastinum and potentially associated pneumothorax, esophageal rupture, or infection within the fascial planes.^2^,^10^ Patients with pneumomediastinum typically present with dyspnea, chest pain, back pain, dysphagia, odynophagia, or Hamman’s sign (a systolic friction rub).^5^,^7^,^10^,^11^

Diagnosis of subcutaneous emphysema and pneumomediastinum is confirmed radiographically. A CT of the chest and neck is the most sensitive test for detecting widespread emphysema and pneumomediastinum.^4^,^5^ If CT is unavailable, use of plain radiographs of the chest and neck will show radiolucent layers of free air.

Subcutaneous emphysema by itself, despite the possibility of leading to local infection, is relatively benign and innocuous, with most cases spontaneously resolving within 2–10 days. These patients can be managed with reassurance, analgesia, and observational telemetry monitoring for cardiac and respiratory efforts. Since infection is rare, antibiotics are not always necessary; nevertheless, they are frequently prescribed. Patients with pneumomediastinum, however, are typically admitted for IV prophylactic, broad-spectrum antibiotics to cover oral aerobes and anaerobes. The possibility of further complications may be decreased by use of sedatives to lower respiratory effort. Additionally, stool softeners, antitussives, and antihistamines to decrease intrathoracic pressure generated from Valsalva, coughing, and nose blowing may be efficacious.

In most cases, the reabsorption of air begins within two to three days, frequently having complete resolution by day 7–10 after onset.^8^,^9^ This process may be hastened with the use of oxygen inhalation through nasal cannula or nonrebreather, which reduces the partial pressure of nitrogen within the blood, ultimately increasing air reabsorption.^10^ In the setting of ED presentation following dental work with a high-pressure drill or syringe, further invasive procedures such as nasoendoscopy, bronchoscopy, esophagoscopy, or barium esophagram are unnecessary.

## CONCLUSION

Cervicofacial and mediastinal emphysema rarely occurs following a common dental procedure such as molar extraction. Most reported cases are localized to the cervicofacial regions; only a few cases have been reported with mediastinal extension. It is imperative that clinicians be able to identify and diagnose complications associated with face or neck swelling following dental procedures as most cases are misdiagnosed and the complications could be fatal.

## Figures and Tables

**Image:**
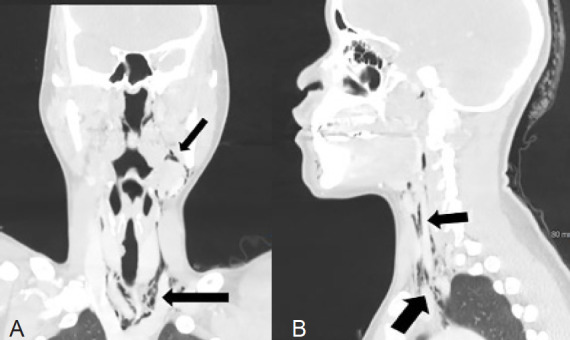
(A) Computed tomography (CT) with intravenous contrast (coronal) demonstrating extension of the subcutaneous emphysema from left lower molar tooth, along both the anterior and posterior triangles extending up to the retrosternal area and into the mediastinum (arrows). (B) CT with intravenous contrast (sagittal) demonstrating extension of the subcutaneous emphysema from the left lower molar tooth into the retropharyngeal space and mediastinum (arrows).
